# Antithrombotic treatment during coronary angioplasty after failed thrombolysis: strategies and prognostic implications. Results of the RESPIRE registry

**DOI:** 10.1186/s12872-017-0636-9

**Published:** 2017-08-01

**Authors:** José M. De la Torre Hernández, Mario Sadaba Sagredo, Miren Telleria Arrieta, Federico Gimeno de Carlos, Elena Sanchez Lacuesta, Juan A. Bullones Ramírez, Javier Pineda Rocamora, Victoria Martin Yuste, Tamara Garcia Camarero, Mariano Larman, Jose R. Rumoroso

**Affiliations:** 1 Servicio de Cardiología, Unidad de Hemodinámica y Cardiología Intervencionista, Hospital Universitario Marqués de Valdecilla, Valdecilla Sur, 1ª Planta, 39008 Santander, Spain; 2 Servicio de Cardiología, H de Galdakao, Vizcaya, Spain; 3 Servicio de Cardiología, H. de Donostia, San Sebastian, Spain; 4 Servicio de Cardiología, H. Clínico de Valladolid, Valladolid, Spain; 5 Servicio de Cardiología, H. la Fé de Valencia, Valencia, Spain; 6 Servicio de Cardiología, H. Carlos Haya de Malaga, Malaga, Spain; 7 Servicio de Cardiología, H. General de Alicante, Alicante, Spain; 8 Servicio de Cardiología, H. Clinic de Barcelona, Barcelona, Spain

**Keywords:** Acute myocardial infarction, Thrombolytic therapy, Angioplasty, Anticoagulation

## Abstract

**Background:**

Thrombolysis is still used when primary angioplasty is delayed for a long time, but 25%–30% of patients require rescue angioplasty (RA). There are no established recommendations for antithrombotic management in RA. This registry analyzes regimens for antithrombotic management.

**Methods:**

A retrospective, multicenter, observational registry of consecutive patients treated with RA at 8 hospitals. All variables were collected and follow-up took place at 6 months.

**Results:**

The study included 417 patients. Antithrombotic therapy in RA was: no additional drugs 22.3%, unfractionated heparin (UFH) 36.6%, abciximab 15.5%, abciximab plus UFH 10.5%, bivalirudin 5.7%, enoxaparin 4.3%, and others 4.7%. Outcomes at 6 months were: mortality 9.1%, infarction 3.3%, definite or probable stent thrombosis 4.3%, revascularization 1.9%, and stroke 0.5%. Mortality was related to cardiogenic shock, age > 75 years, and anterior location. The stent thrombosis rate was highest with bivalirudin (12.5% at 6 months). The incidence of bleeding at admission was high (14.8%), but most cases were not severe (82% BARC ≤2). Variables independently associated with bleeding were: femoral access (OR 3.30; 95% CI 1.3–8.3: *p* = 0.004) and post-RA abciximab infusion (OR 2.26; 95% CI 1.02–5: *p* = 0.04).

**Conclusions:**

Antithrombotic treatment regimens in RA vary greatly, predominant strategies consisting of no additional drugs or UFH 70 U/kg. No regimen proved predictive of mortality, but bivalirudin was related to more stent thrombosis. There was a high incidence of bleeding, associated with post-RA abciximab infusion and femoral access.

## Background

Primary angioplasty is the treatment of choice in acute myocardial infarction if it can be performed within 120 min of first medical contact. Fibrinolysis is recommended in patients who have no contraindications if primary angioplasty cannot be performed within 120 min of first medical contact [1, 2]. This time shortens to 90 min in the case of infarctions less than two hours old with extensive territory at risk (anterior infarction). A potential indication for fibrinolysis might be patients presenting within three hours of symptom onset, in whom primary angioplasty cannot be performed within the first 60 min. Fibrinolysis is effective in these patients, albeit at the cost of a slight increase in intracranial bleeding [3]. However, in 25%–30% of cases fibrinolysis fails to achieve reperfusion, and patients have to undergo urgent catheterization and rescue angioplasty (RA) [4, 5]. This has been shown to improve the prognosis compared with a conservative approach or repeat fibrinolysis [6–9].

Patients who require RA are exposed to various antiplatelet drugs and anticoagulants, as well as previous fibrinolytic therapy, which explains the high risk of bleeding observed, of up to 25% [6–9]. Clinical guidelines contain no clear regimens or specific recommendations for the management of antithrombotic therapy in RA [1, 2, 9].

This rescue angioplasty registry was a multicenter observational study designed to analyze the different antiplatelet and anticoagulation regimens used during the procedure and, more importantly, the ischemic and bleeding complications associated with them.

## Methods

The RESPIRE (Registro ESpañol de anticoagulacion en angioPlastIa de REscate) registry is a multicenter prospective study involved 8 Spanish hospitals. All consecutive patients who underwent RA following failed fibrinolysis between January 2012 and December 2013 were included in the study.

The decision to proceed to RA was made by the physicians in charge of each particular case. In general, however, it was indicated after failed fibrinolysis, defined as <50% reduction in ST elevation 60 min after administration of the fibrinolytic, with or without chest pain. Presentation in cardiogenic shock was not excluded.

All data were entered into an anonymized database, including past cardiovascular history, clinical data, CRUSADE bleeding score, details of the procedure, and events at discharge and one and six months post-procedure. The registry was approved by the respective ethics committees of the participating centers and was in compliance with the Helsinki Declaration.

### Study endpoints and definitions

Major adverse cardiac events were defined as follows: a) death as all-cause mortality; b) cardiac death as mortality due to heart conditions such as infarction, heart failure or stent thrombosis, including sudden death of undefined origin; c) myocardial infarction (MI) if detailed criteria were met. The criteria for MI were: 1) detecting a rise and fall in cardiac biomarkers (preferably troponin) with at least one value above the 99th percentile upper reference limit, together with signs of myocardial ischemia with at least one of the following symptoms: chest pain, electrocardiographic abnormalities (new-onset ST-T changes or new-onset left bundle branch block) or onset of pathological Q waves, new-onset regional wall motion or perfusion abnormalities; 2) sudden death with cardiac arrest, often preceded by symptoms suggestive of ischemia, accompanied by ST elevation of presumably new onset or new-onset left bundle branch block, or angiographic or post-mortem evidence suggesting recent thrombus (if death occurred before blood samples could be obtained or before cardiac markers appeared in the blood); and 3) pathological signs of acute infarction. Revascularization was defined as any angioplasty procedure or coronary revascularization surgery. Stent thromboses were classified according to the Academic Research Consortium (ARC) classification, and bleeding was categorized in accordance with the Bleeding Academic Research Consortium (BARC) classification [10, 11].

### Statistical analysis

Continuous parameters are expressed as mean ± standard deviation or median (interquartile range). Categorical variables are reported as percentages. Categorical variables were compared using the chi-square test or Fisher’s exact test. A Kolmogorov-Smirnov test was performed to evaluate the normal distribution of continuous variables. Continuous variables were compared using ANOVA or the Kruskall-Wallis test, depending on their distribution. A multivariate logistic regression model was constructed to establish independent predictors of events during hospitalization. Statistical analysis was two-tailed, and statistical significance was taken as *p* < 0.05. Analysis was performed using the statistical package SPSS 15.0.

## Results

**Fig. 1:**
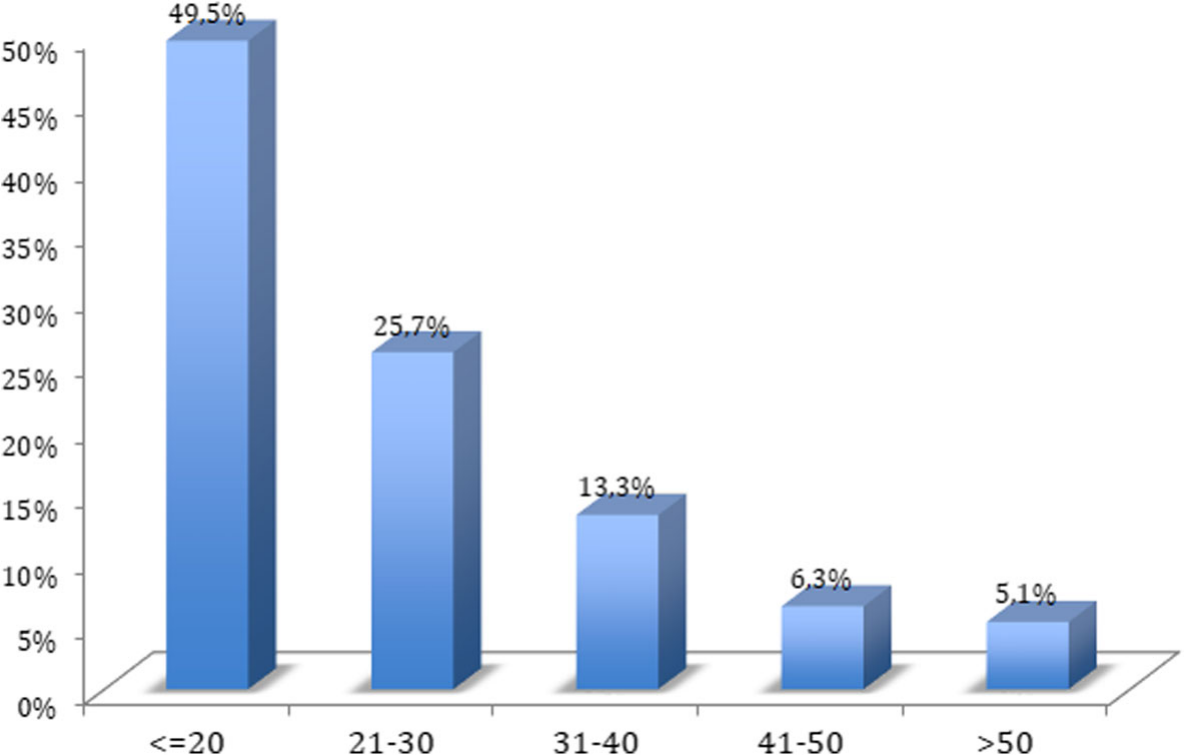
CRUSADE bleeding score in the study population

Four hundred and seventeen consecutive patients with acute myocardial infarction who underwent RA were included. Their clinical features are described in Table 1. Elapsed time between thrombolysis and RA was 219 ± 165 min with a median of 170 (IQ range 132–244). The pre-catheterization thrombolysis and anticoagulation regimen was, with few exceptions, tenecteplase (TNK-tPA) plus enoxaparin, and the pre-catheterization antiplatelet regimen was aspirin plus clopidogrel 300 mg in the vast majority of cases (85.6%). Most patients (88.5%) had a CRUSADE score of less than 40 (Fig. 1).

**Table 1:** Clinical characteristics

	*N* = 417
Age (years)	61.6 ± 12
Females	59 (14.1%)
Weight (kg)	80 ± 12.8
Height (cm)	169.4 ± 7.6
Hypertension	208 (50)
Diabetes	86 (20.6)
Smoking	242 (58)
Dyslipidemia	192 (46)
Previous stroke	17 (4)
Peripheral vascular disease	17 (4)
Previous infarction	41 (9.8)
HR at admission (bpm)	76.9 ± 19.2
SBP at admission (mmHg)	124.8 ± 27.1
Creatinine (mg/dL)	0.99 ± 0.5
Baseline hematocrit (%)	40 ± 8.6
Ejection fraction (%)	49.3 ± 12.2
Pain onset to thrombolysis (minutes)	147 ± 111
	115 (80–175)
Thrombolysis to PCI (minutes)	219 ± 165
	170 (132–244)
Thrombolytic regimen	
TNK-tPA + enoxaparin	391 (93.7)
TNK-tPA + fondaparinux	21 (5)
TNK-tPA + unfractionated heparin	5 (1.2)
Pre-RA Killip class	
Killip I	318 (76.2)
Killip II	48 (11.5)
Killip III	17 (4.1)
Killip IV	34 (8.2)

Qualitative variables are shown as n (%) and quantitative variables as mean ± standard deviation, but times are also shown as median and interquartile range

*HR* heart rate, *PCI* percutaneous coronary intervention, *SBP* systolic blood pressure, *TNK-tPA* tenecteplase, *UFH* unfractionated heparin

Table 2 shows the approach used for catheterization and the antithrombotic regimen used to perform RA. The anticoagulation regimens used during the procedure varied greatly, but consisted predominantly of not using any additional drugs or using unfractionated heparin (UFH) at 70 U/kg. IIb/IIIa receptor inhibitors (abciximab) were used alone or in combination with UFH in 26% of patients, with or without post-procedural infusion. Table 2 also shows pre- and post-RA flow status. It should be noted that 43% had a TIMI flow of 2 or 3 when the procedure started, and TIMI flow 3 had been achieved in 89.4% of patients when it ended.

**Table 2:** Procedural characteristics: access site, antithrombotic therapy, and arterial flow status

		*N* = 417
Femoral access		217 (52)
Radial access		200 (48)
Antithrombotic therapy		
No additional treatment		93 (22.3)
UFH		153 (36.6)
UFH 70 U/kg	115	
UFH 100 U/kg	38	
Enoxaparin		18 (4.3)
Enoxaparin 0.35 mg/kg	3	
Enoxaparin 0.5 mg/kg	15	
Bivalirudin		24 (5.7)
Bivalirudin in cath lab	10	
Bivalirudin in cath lab +4 h	14	
Abciximab		65 (15.5)
Abciximab bolus	12	
Abciximab bolus plus infusion	53	
Abciximab plus UFH		44 (10.5)
Abciximab bolus	18	
Abciximab bolus plus infusion	26	
Other regimens		20 (4.7)
Baseline flow		
TIMI 0		196 (47)
TIMI 1		44 (10.5)
TIMI 2		66 (15.8)
TIMI 3		111 (26.6)
Final flow		
TIMI 0		13 (3.1)
TIMI 1		9 (2.2)
TIMI 2		22 (5.3)
TIMI 3		373 (89.4)

*UFH* unfractionated heparin

Table 3 shows the various subgroups according to antithrombotic treatment regimen. Significant differences exist for many of the variables examined. Younger patients were treated with abciximab alone, and patients of lower body weight received no additional therapy. This subgroup, in whom no new antithrombotic drugs were added, included patients with higher systolic blood pressure and higher creatinine values. The longest delay between thrombolysis and RA was seen in the subgroup treated with UFH plus abciximab. As regards the procedure, radial access was uncommon in patients given no additional therapy and those treated with abciximab alone. Baseline coronary flow was more conserved in patients with no additional therapy, and less so in those treated with combined UFH and abciximab. Flow restoration was consequently somewhat less successful in the latter group, and more stents had to be implanted. These data indicate regimen selection on the basis of baseline flow status (thrombus burden) and bleeding risk profile. The prescribed time period for dual antiplatelet therapy was 12 months in all patients but no data on compliance was available.

**Table 3:** Clinical features and procedural characteristics by antithrombotic treatment group

	Nothing *n* = 93	UFH *n* = 153	UFH + Abcx *n* = 44	Abcx *n* = 65	Bivalirudin *n* = 24	Enoxaparin *n* = 18	p
Age (years)	61 ± 13	60.3 ± 14	61.3 ± 11	55.4 ± 4	61 ± 11	63.9 ± 9	<0.001
Females	15 (16)	21 (13.7)	6 (13.6)	7 (10.7)	1 (4.2)	4 (22)	0.2
Weight (kg)	77.5 ± 10	81 ± 13	81 ± 12	80 ± 13	81 ± 12	81 ± 17	0.01
Hypertension	51 (54.8)	74 (48.3)	18 (40.9)	30 (46)	14 (58.3)	9 (50)	0.2
Smoking	50 (53.7)	81 (53)	24 (54.5)	43 (66)	17 (70.8)	9 (50)	0.1
Diabetes	23 (24.7)	34 (22.2)	3 (6.8)	13 (20)	4 (16.6)	5 (27.7)	0.04
Dyslipidemia	47 (50.5)	69 (45)	15 (34)	31 (47.6)	15 (62.5)	8 (44)	0.03
Previous stroke	3 (3.2)	8 (5.2)	1 (2.2)	2 (3)	0	1 (5.5)	0.5
Previous infarction	7 (7.5)	14 (9.1)	1 (2.2)	11 (16.9)	4 (16.6)	3 (16.6)	0.03
Peripheral vascular disease	5 (5.4)	6 (3.9)	2 (4.5)	1 (1.5)	1 (4.2)	1 (5.5)	0.05
Coronary surgery	1 (1)	2 (1.3)	0	1 (1.5)	0	0	0.4
SBP at admission (mmHg)	135 ± 28	123 ± 27	117 ± 22	127 ± 24	113 ± 30	120 ± 24	<0.001
HR at admission (bpm)	78 ± 19	77.5 ± 19	75.7 ± 19	79 ± 20	74 ± 19	67 ± 15	0.02
Creatinine (mg/dL)	1.1 ± 0.6	0.9 ± 0.3	1 ± 1	1 ± 0.3	0.9 ± 0.2	1 ± 0.2	<0.001
Baseline hematocrit (%)	40 ± 10	41.5 ± 5	41 ± 6	37 ± 12	40.8 ± 7	38.3 ± 11	<0.001
Anterior infarction	50 (53.7)	62 (40.5)	22 (50)	31 (47.6)	11 (45.8)	9 (50)	0.05
Ejection fraction (%)	46.6 ± 11	50.8 ± 12	51 ± 14	47 ± 13	50 ± 13	50.7 ± 11	0.02
Pain onset to thrombolysis	120	121	105	105	120	88	0.09
Thrombolysis to rescue	167	164	205	168	182	175	0.003
TNK-tPA + enoxaparin	89 (95.6)	144 (94)	39 (88.6)	60 (92.3)	21 (87.5)	17 (94.4)	0.2
Killip class III-IV	12 (13)	12 (7.8)	5 (11.4)	11 (17)	5 (20.8)	0	0.08
Radial access	19 (20.4)	100 (65.3)	31 (70.4)	11 (17)	11 (45.8)	17 (94.4)	<0.001
Baseline TIMI	1.7 ± 1.3	1.4 ± 1.3	0.57 ± 1	0.78 ± 1	0.75 ± 1.2	16 ± 1.3	<0.001
Final TIMI	2.8 ± 0.7	2.9 ± 0.5	2.75 ± 0.7	2.7 ± 0.6	2.8 ± 0.6	3 ± 0	0.01
Baseline TIMI 0–1	38 (40.8)	77 (50.3)	36 (81.8)	45 (69.2)	18 (75)	6 (33.3)	<0.001
Final TIMI 3	86 (92.5)	143 (93.4)	35 (79.5)	51 (78.4)	22 (91.6)	18 (100)	0.003
Thrombus aspiration	23 (24.7)	56 (36.6)	28 (63.6)	38 (58.4)	13 (54.2)	6 (33.3)	0.001
Number of stents	1.2 ± 0.6	1.3 ± 0.7	1.37 ± 0.9	1.27 ± 0.8	1.08 ± 0.6	1.1 ± 0.5	0.1
Multivessel disease	41 (44)	48 (31.4)	18 (40.9)	27 (41.5)	13 (54.2)	8 (44)	0.04
2nd PCI	7 (7.5)	18 (11.7)	7 (16)	6 (9.2)	5 (20.8)	0	0.1
Clopidogrel load 300 mg	85 (91.4)	126 (82.3)	37 (84)	55 (84.6)	19 (79.2)	11 (61)	0.002

Qualitative variables are shown as n (%) and quantitative variables as mean ± standard deviation, except times, which are shown as medians

*Abcx* abciximab, *HR* heart rate, *PCI* percutaneous coronary intervention, *SBP* systolic blood pressure, *TNK-tPA* tenecteplase, *UFH* unfractionated heparin

**Fig. 2:**
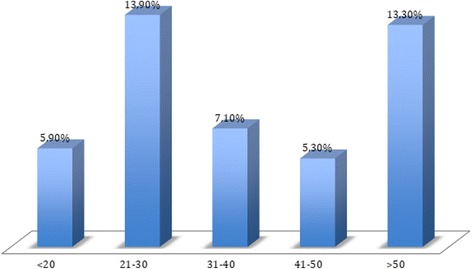
BARC >1 bleeding rate according to CRUSADE bleeding score

The 6-month events rate in the overall population is shown in Table 4. The procedure mortality rate was 2.4%, and the in-hospital mortality rate 7.4%. As bleeding was the most common event, and is particularly important in this context, a detailed classification of bleeding that occurred during hospitalization is shown in Table 5. The vast majority of bleeding events were not severe, but 11 (2.6%) high-grade bleeding episodes (BARC >2) occurred. The risk of BARC >1 bleeding was significantly higher with the femoral approach than with radial access: 13.7% versus 4.3% (*p* = 0.001). Most patients had a low initial bleeding risk based on their CRUSADE score, as shown in Fig. 1. The CRUSADE score was not correlated with the incidence of BARC >1 bleeding, as illustrated in Fig. 2.

**Table 4:** Events at 6 months

	*n* = 417
Death	38 (9.1%)
Cardiac death	33 (7.9%)
Infarction	14 (3.3%)
Definite/probable thrombosis	18 (4.3%)
BARC >1 bleeding	40 (9.6%)
BARC >2 bleeding	14 (3.3%)
Brain hemorrhage	5 (1.2%)
Revascularization	8 (1.9%)
Stroke	2 (0.5%)

*BARC* Bleeding Academic Research Consortium

**Table 5:** Bleeding during hospitalization

	*N* = 62
BARC 1	26 (6.2)
BARC 2	25 (6.0)
BARC 3a	2 (0.5)
BARC 3b	2 (0.5)
BARC 3c	4 (0.9)
BARC 4	1 (0.2)
BARC 5	2 (0.5)

Clinical outcome by treatment subgroup is shown in Table 6, which lists all events detected in 6 months. There were no differences in mortality, although this tended to be higher in the group treated with combination UFH plus abciximab, most likely because this group had more adverse features, as mentioned above. The incidence of infarction and thrombosis was significantly higher in the bivalirudin group. As regards bleeding, patients treated with abciximab alone had a significantly higher risk, probably because of a lower rate of radial access use (17%) and greater use of post-procedure infusion (81.5%). These values were 70.4% and 59% respectively in the abciximab plus UFH group. The bleeding rate in the group given an abciximab bolus but no infusion was notably lower, in numerical terms, than in patients given a bolus plus infusion (BARC >1 of 10% versus 15%, and BARC >2 of 3.3% versus 6.3%), and there were fewer ischemic events (0% thrombosis/infarction rate versus 7.5%).

**Table 6:** Events at 6 months by antithrombotic treatment group

	Nothing *n* = 93	UFH *n* = 153	UFH + Abcx *n* = 44	Abcx *n* = 65	Bivalirudin *n* = 24	Enoxaparin *n* = 18	p
Death	9 (9.6)	10 (6.5)	5 (11.4)	5 (7.7)	2 (8.3)	1 (5.5)	0.4
Cardiac death	8 (8.6)	9 (5.9)	5 (11.4)	4 (6.1)	2 (8.3)	0	0.3
Infarction	1 (1)	3 (1.9)	2 (4.5)	1 (1.5)	3 (12.5)	0	0.04
Def./probable thrombosis	2 (2.1)	7 (4.6)	1 (2.2)	2 (3)	3 (12.5)	0	0.08
BARC >1 bleeding	10 (10.7)	10 (6.5)	3 (6.8)	12 (18.5)	1 (4.2)	2 (11.1)	0.01
BARC >2 bleeding	5 (5.4)	2 (1.3)	0	6 (9.2)	0	1 (5.5)	0.01
Brain hemorrhage	3 (3.2)	0	0	1 (1.5)	0	1 (5.5)	0.1
Revascularization	0	3 (1.9)	1 (2.2)	2 (3)	0	0	0.3
Stroke	1 (1)	0	0	1 (1.5)	0	0	0.7

*Abcx* abciximab, *BARC* Bleeding Academic Research Consortium, *UFH* unfractionated heparin

In multivariate analysis for predictors of BARC >1 bleeding, femoral access and post-RA abciximab infusion were identified as predictors, and a strong trend was seen for age > 75 years (Table 7). The higher bleeding risk conferred by post-RA abciximab infusion was very obvious in the case of femoral access (24% BARC >1 bleeding), but not if the radial approach was used (4%).

**Table 7:** Independent predictors of BARC >1 bleeding

Variable	OR	95% CI	P
Femoral access	3.30	1.3–8.3	0.004
Abciximab infusion	2.26	1.02–5	0.04
Age > 75 years	2.3	0.95–5.62	0.07

Table 8 shows the variables found to be independent predictors of mortality, which were cardiogenic shock, age > 75 years, and anterior infarction.

**Table 8:** Independent predictors of mortality

Variable	OR	95% CI	P
Cardiogenic shock	60.7	13.5–272	<0.001
Age > 75 years	6.5	1.8–23.3	0.003
Anterior location	6.2	1.5–24.8	0.005

## Discussion

The main findings of the RESPIRE registry are: a) The thrombolysis regimen, and the antiplatelet and anticoagulation regimens associated with it, were fairly standardized; b) There was a long delay between thrombolysis and RA; c) RA did not employ radial access in most cases, even though the increased bleeding risk in these patients is well known; d) The anticoagulation and antiplatelet regimens used in RA varied markedly, with a predominance of no additional drugs or UFH at 70 U/kg; e) Abciximab was used in just over a quarter of patients; f) Mortality was relatively higher than in primary angioplasty series, and was related to cardiogenic shock, age > 75 years, and anterior infarction; g) The incidence of bleeding was high, but most bleeding episodes were not severe; post-RA abciximab infusion and femoral access were associated with greater bleeding risk.

The superiority of primary angioplasty over fibrinolysis when performed soon enough is well established. Infarction care systems based on primary angioplasty have managed to reduce the overall mortality rate substantially, not just because of the advantages of primary angioplasty, but also due to the consequent large reduction in the non-reperfusion rate [1, 2]. Despite this, fibrinolytic therapy is still required in a certain proportion of patients, specifically those presenting at sites with no catheterization laboratory, and in whom the estimated time to primary angioplasty exceeds 120 min. Nevertheless, thrombolysis is associated with a reperfusion failure rate and a certain early reocclusion rate. The proportion of patients requiring RA is about 25%–30% [4, 5]. The indication for RA in these cases is well established, because of the advantages it provides in terms of patient prognosis, compared with a conservative approach or repeat fibrinolysis [6–8].

The scenario of rescue angioplasty after fibrinolysis involves an unresolved thrombotic event and high bleeding risk because of previously administered antiplatelet, anticoagulant and thrombolytic therapy. There are no indications or recommendations in the guidelines regarding technical details or antithrombotic therapy as an adjunct to RA. The guidelines establish antithrombotic regimens for patients receiving fibrinolysis, but not for those undergoing rescue angioplasty [1, 2, 9]. This absence of recommendations for interventions of this type is due to a lack of evidence from trials, with only registries of limited size available.

With regard to access site choice, a large RA registry in the United States reported a low rate of radial access use (14.2%). In propensity-matched analysis, the radial approach was associated with a significantly lower bleeding risk, with no influence on mortality [12].

In this respect, studies evaluating the safety of administering IIb/IIIa inhibitors (especially abciximab) are particularly worthy of note. One of these was a small randomized study involving 89 patients who underwent RA, 44 treated with abciximab and 45 not [13]. The 6-month results showed a lower incidence of events in the treated group, with no increase in bleeding. These results have been replicated in some registries, [14] but other larger ones have found no reduction in events with abciximab when clopidogrel pretreatment was given [15, 16]. As regards bleeding risk, whereas this is clearly increased with abciximab in some registries, [17] in others this is not the case [14, 16, 18]. All registries clearly demonstrate the prognostic importance of shock and of obtaining adequate flow, as well as the need to reduce bleeding complications [15, 19–21]

Our study reflects this lack of consensus regarding antithrombotic therapy in rescue angioplasty, because although fibrinolysis treatment was very uniform, there was great variability in terms of anticoagulant and IIb/IIIa inhibitor use in RA. We also found a lack of correlation between CRUSADE score and the incidence of bleeding. The context of RA and the particular treatment combinations seem to make this score less valid.

The rate of major bleeding in recent fibrinolysis studies was 7.5% in STREAM [3] and 1.7% in FAST-MI [22]. In the REACT study, the major bleeding rate was 0.6% and the minor bleeding rate 22.9% [7]. In this study, heparin sodium was administered in fibrinolysis and during rescue angioplasty, and abciximab was used in 43.4% of rescue angioplasties. Among patients who experienced bleeding, 69% had been given abciximab, although this difference was not statistically significant compared with the rest of the patients. As regards mortality, in the RA arm in the REACT study, overall mortality at 6 months was 6.2%, and age and diabetes were identified as predictors of mortality [7]. In the MERLIN study, one-month mortality in the RA arm was 9.8%, and anterior infarction was the only predictor of mortality [6]. The mortality rate in these studies is comparable to the rate found in our study, although cardiogenic shock was an exclusion criterion in the trials. These results suggest potencial relevant differences in patients characteristics as it is commonly observed between randomized trials and observational registries, furthermore if performed in different time. Anterior infarction and age were likewise predictive factors for mortality.

Attention must be drawn to the time elapsed between thrombolysis and RA. Bearing in mind that the indication arose because of ineffective thrombolysis, a median of 170 min is very high, because if the indication should be established by 60 min post-thrombolysis, this represents a delay of almost another 2 h until RA. It should be noted that radial access was not used in most cases in this registry, despite the known higher bleeding risk of these patients. In fact, femoral access combined with abciximab use, especially if post-RA infusion was involved, raised the bleeding risk considerably. Finding fewer bleeding episodes without the disadvantage of more ischemic events when abciximab was used in bolus form only, compared with additional infusion use, is thought-provoking. It is also interesting to note the 42% thrombus aspiration rate, despite previous thrombolysis. Although no antithrombotic regimen proved to be an independent predictor of ischemic events, bivalirudin use was associated with the highest stent thrombosis rate.

### Limitations

The most obvious limitation is non-randomization for the different antiplatelet and anticoagulation regimens employed in RA. There was likewise no randomization for other aspects of the procedure, such as radial or femoral access. A randomized study would be particularly difficult in this context. In fact, apart from the occasional small study addressing abciximab use, no randomized trials were attempted during the years when RA was much more common, and they are even less likely now. This was a multicenter, consecutive registry, which makes the results more valuable and robust. Nevertheless, between-group comparisons are clearly subject to bias. The size of the groups precludes adjustment by treatment propensity score. The sample could have been larger, but this would have been difficult because the RA caseload has decreased greatly with the more widespread use of primary angioplasty. Data were not monitored off-site, because this was beyond our means. The comparability of results with previous trials on the topic, specifically with the MERLIN trial, [6] could be limited by differences in population profile. As mentioned before, the 30-day mortality was similar between our registry and this trial, despite the exclusion of cardiogenic shock in the later. The observational and more recent nature of our registry may account for these differences.

## Conclusions

Antithrombotic treatment regimens during rescue angioplasty procedures vary greatly, although not adding any new drugs, and using UFH 70 U/kg alone, are the most predominant. The study did not identify any antithrombotic treatment variable independently related to mortality to any significant extent, but abciximab infusion and femoral access were associated with more bleeding risk. Therefore, in situations of rescue angioplasty, combining these two strategies should be avoided as far as possible. Considering the incidence of events in this observational study, we think it would be advisable for a prospective study to be conducted, to try to establish the safest, most effective antithrombotic treatment regimen in rescue angioplasty.

## Abbreviations

BARC: Bleeding academic research consortium
MI: Myocardial infarction
RA: Rescue angioplasty
UFH: Unfractionated heparin
